# Chemical Composition and Nutritional Value of Three *Sonchus* Species

**DOI:** 10.1155/2022/4181656

**Published:** 2022-03-03

**Authors:** Galdino Xavier de Paula Filho, Tibério Fontenele Barreira, Helena Maria Pinheiro-Sant'Ana

**Affiliations:** ^1^Department of Education, Federal University of Amapá, Macapá 68903-419, Brazil; ^2^Department of Plant Science, State University of North Fluminense Darcy Ribeiro, Campos dos Goytacazes 28013-602, Brazil; ^3^Department of Nutrition and Health, Federal University of Viçosa, Viçosa 36571-000, Brazil

## Abstract

Species of unconventional food plants of the genus *Sonchus* are widely consumed in rural populations living in the Brazilian Atlantic Forest. This study investigated the nutritional composition of *S. oleraceus*, *S. asper*, and *S. arvensis* species. The centesimal composition was investigated according to the norms of the Association of Official Analytical Chemists, the occurrence and concentration of carotenoids and vitamins through High-Performance Liquid Chromatography, and minerals with the aid of atomic emission spectrometry in inductively coupled plasma. There was no significant difference between the water content found in the three species. However, *S. asper* showed higher concentrations of lipids (1.32 g/100 g), carbohydrates (0.34 g/100 g), total carotenoids (5.58 mg/100 g), and Ca (96.25 mg/100 g), while *S. arvensis* had the highest concentration of vitamins E (72.98 *μ*g/100 g) and K (604.85 mg/100 g). *S. oleraceus* showed higher concentrations of Fe (23.74 mg/100 g). Statistically, fibers and ash presented the same proportions in *S. asper* and *S. arvensis*, as well as proteins in *S. oleraceus* and *S. asper* species. The availabilities of these vegetables together with their high nutritional value are important factors that contribute to ensuring food security for families that have these species in their diet.

## 1. Introduction

Unconventional food plants are species with spontaneous spread, receive no handling, and are found along roads, in orchards, in the middle of a pasture, and other agricultural crops. Medicinal plants have become a worldwide topic drawing an impact on world health. Herbal medicine has played a crucial role in the maintenance of the healthcare system of the wide population throughout the world. The major issue for the modern healthcare industry is to overcome pathogen resistance to antimicrobial agents and diseases caused by oxidative stress. Silver nanoparticles are also effective antiangiogenesis, anti-inflammatory, antiplatelet, and antiviral agents [1, 2]. They are food plant species that, with the beginning of the modernization in farming, have not undergone the process of genetic improvement aiming at their technical production. Nevertheless, they are found in all biomes in Brazil, and some of them predominate in certain regions [3].

These species include leafy vegetables, fruits, rhizomes, and flowers that have historically been part of the diet of the rural population and in many cases are the only available resources for their food [1, 4]. Some studies point to the hypothesis that these species may be nutritionally rich [5–7]. However, for many of them, there are still no studies on their nutritional composition [8].

The Atlantic Forest represents the third largest biome in Brazil in terms of territorial extension and fauna and flora biodiversity. It is also the most populous, concentrating 60% of the Brazilian population. However, it is one of the most devastated, with only 11.6% of its original vegetation cover, and the remaining percentage is protected by law [9, 10]. Apart from this percentage, there are the urbanized areas (cities and towns) and those used for farming activities, characterized by intensive cattle ranching, coffee growing (*Coffea canephora* L.), eucalyptus monoculture (*Eucalyptus* spp.), and areas of family farmers, intended for the production of annual crops, vegetable, and fruit growing [11]. In the midst of this scenario, together with other agricultural crops and forest fragments, it can be found a wide diversity of nonconventional food species highly demanded for the food consumption of the local population [2–8].

Among these species, some of the botanical genus *Sonchus* stand out. This species is locally known as common sow thistle (*Sonchus oleraceus* L.), prickly sow thistle (*Sonchus asper* (L.) Hill), and smooth sow thistle (*Sonchus arvensis* L.). These species belong to the botanical family *Asteraceae*. They are eaten mainly sautéed with sauces and broths [2]. From a physiological point of view, they are species of erect herbaceous size and annual cycle; their stem is hollow and little branched and also has simple, sessile leaves, with membranous blade with an auricular base and 6 to 17 cm long [12].

These species are originally from Europe, but they are found throughout the American continent, Africa, Asia, Australia, and New Zealand [13]. In addition to their food use, they are known for their antioxidant activity, being used in phytotherapy and their allelopathic potential used against invasive plants in agriculture [12, 14, 15]. However, in countries like Pakistan, Iran, and Russia, these species are considered weeds [16–18].

In Brazil, these species are found in native environments, especially in the South and Southeast regions of the country, being used for food and medicinal purposes [2, 12, 19]. However, some sources regard them as invasive plants in agriculture [20, 21]. As food, these species are eaten as salads and sautéed and cooked with other foods [2, 12].

These species are part of the food habits of population groups in several countries, consumed *in natural*, or submitted to cooking. They are considered sources of dietary fiber and vitamins and strongly contribute to the food security strategies of thousands of people [22–24].

Thus, the objective of this study was to investigate the concentration of macronutrients and fibers and carotenoids, vitamins (A and E), and minerals in three unconventional food species of the genus *Sonchus* (*S. oleraceus* L., *S. asper* (L.) Hill, and *S. arvensis* L.), eaten by rural populations, in samples collected in wild environments.

## 2. Materials and Methods

### 2.1. Sample Collection and Preparation

**Figure 1:**
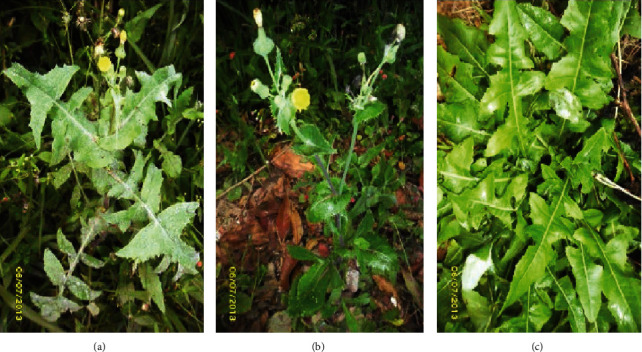
S*. oleraceus* (a), *S. asper* (b), and S. arvensis (c).

The species common sow thistle (*S. oleraceus* L.), prickly sow thistle (*S. asper* (L.) Hill), and smooth sow thistle (*S. arvensis* L.) (Figure 1) were collected in their adult stage, obtained in native environments, from spontaneously propagated plants found in the rural area of Viçosa, State of Minas Gerais (MG), Brazil (south latitude 20°44′ and west longitude 42°50′40^″^). The species analyzed in this study were identified in herbaria in the region; they have the following identifications: *S. oleraceus* L., IAC 6669; *S. asper* (L.) Hill, IAC 6668; and *S. arvensis* L., RB 428940.

Sampling was carried out in five replications, composed of 500 g of vegetables in each replicate, and collected in five different rural communities. After collection, the samples were immediately transported to the Vitamin Analysis Laboratory of the Department of Nutrition and Health (DNS) of the Federal University of Viçosa (UFV), in plastic bags protected against light. Carotenoid analyses were performed within 36 h after collection, and vitamin E analyses were performed within 72 h.

The species were washed under running water to remove impurities and dried with paper towels. Next, the leaves and stems showing partial or complete light-yellow color and tender texture, in addition to those attacked by insects, were removed. Later, the edible parts were homogenized in a food processor (Faet Multipratic, MC5), packed in plastic polyethylene bags covered by aluminum foil and stored at −18 ± 1°C. For the analysis of macronutrients, fibers, and minerals, the samples were dehydrated in an oven with forced air circulation at 65 ± 1°C, for 72 h, and stored in plastic polyethylene bags, until analysis.

### 2.2. Chemical Reagents and Equipment Used in the Experiment

For the extraction of carotenoids and vitamin E, the following analytical-grade reagents were used: acetone and petroleum ether (Vetec, Brazil). The following HPLC-grade reagents were used for the analysis: acetone, hexane, isopropanol, ethyl acetate, methanol and acetonitrile (Tedia, Brazil), and glacial acetic acid (Vetec, Brazil).

The vitamin E standards (*α*-, *β*-, *γ*-, and *δ*-tocopherols and tocotrienols) were acquired from Calbiochem®, EMD Biosciences, Inc. (USA). The *α*-carotene and *β*-carotene patterns were isolated from concentrated carrot extract; *β*-cryptoxanthin and lycopene were isolated from tomato and papaya extracts, respectively, by open-column chromatography [25].

For the filtration of the samples, it was used filter paper no. JP41 J. (Prolab, Brazil), HV Millex filter units, in polyethylene with 0.45 *μ*m of porosity (Millipore, Brazil) and 3 ml sterilized disposable syringes (TKL, China).

### 2.3. Chemical Analysis

The analyses of moisture, ash, proteins, lipids, and total dietary fiber were determined in three replications [26]. Moisture was determined in an oven at 65 ± 1°C, for 72 h, and for ash analysis, a muffle (QUIMIS) was used at 550°C, for 6 h. The protein concentration was determined using the micro-Kjeldhal method, in which the crude protein was calculated by multiplying the nitrogen (N) content by 6.25 [26]. The total dietary fiber concentration was determined using the nonenzymatic gravimetric method [26].

Total carbohydrates were determined through the difference between 100 and the sum of protein fractions, lipid, moisture, fiber, and ash [27]. The total energy value (TEV) was estimated considering the conversion factors of 4, 9, and 4 kcal per g for carbohydrates, lipids, and proteins, respectively [28].

For the extraction of the components of vitamin E (*α*-, *β*-, *γ*-, and *δ*-tocopherols and tocotrienols), 10 g of the sample was weighed in a semianalytical balance, and 4 ml of heated ultrapure water (80 ± 1°C) was added. After that, 10.0 ml of isopropanol, 1.0 ml of hexane containing 0.05% BHT, and 5 g of anhydrous sodium sulfate were added. Next, 25 ml of the extraction solvent mixture (hexane: ethyl acetate, 85 : 15 *v*/*v*) was added. After these procedures, the sample was ground in a microgrinder at an average speed of 1 min^−1^. Once crushed, the samples were vacuum-filtered in a Büchner funnel using filter paper and keeping the residue in the extraction tube. The extraction step was repeated by adding 5 ml of isopropanol and 30 ml of the solvent mixture, with subsequent homogenization and vacuum filtration. Then, the extract was concentrated on a rotary evaporator at 70 ± 1°C for about 2 min^−1^ and transferred to a volumetric flask, and the volume completed to 25.0 ml with solvent mixture. After extraction, 5.0 ml aliquots of the vegetable extract were evaporated under nitrogen gas flow, redissolved in 2.0 ml of HPLC grade-hexane, and filtered using filter units with 0.45 *μ*m porosity [29].

The analyses were performed in a High-Performance Liquid Chromatography system (CLAE) (Shimadzu, SCL 10 AD VP) coupled to a fluorescence detector (RF-10A XL) (290 nm excitation and 330 nm emission), consisting of a high-pressure pump with a valve for low pressure quaternary gradient (LC-10 AD VP), automatic injector with a 50 *μ*l sampling loop (SIL-10AF), and Luna column (Phenomenex, 100A, 250 × 4.62 mm, 5 *μ*m), and equipped with a guard column (Phenomenex Si100, 4 mm × 3 mm). The mobile phase was composed of hexane:isopropanol:glacial acetic acid (98.9 : 0.6 : 0.5, *v*/*v*/*v*), flow of 1.0 ml min^−1^ [29].

In the extraction of carotenoids (*α*-carotene, *β*-carotene, *β*-cryptoxanthin, and lycopene), the method proposed by Rodriguez-Amaya et al. [30] was used. Therefore, 5 g of each vegetable was weighed in a semianalytical balance, added with 60 ml of cooled acetone (divided into three volumes of 20 ml), homogenized in a microgrinder for approximately 5 minutes, and vacuum-filtered in a Büchner funnel using filter paper. Next, the filtrate was transferred, in three fractions, to a separation funnel containing 50 ml of cooled petroleum ether; each fraction washed three times with distilled water for a complete removal of the acetone. Anhydrous sodium sulfate was added to the petroleum ether extract to remove any water residue that might have remained and that could impair the evaporation of the material. Afterwards, the ether extract was concentrated using a rotary evaporator at a temperature of 35 ± 1°C and transferred to a 25.0 ml volumetric flask, and the volume was completed with petroleum ether. For the analysis, 5.0 ml aliquots of vegetable extracts were evaporated under nitrogen gas flow; the dry residue was redissolved in 2.0 ml of HPLC grade-acetone. The extracts were filtered in filter units with a porosity of 0.45 *μ*m, with 30 *μ*l injected into the chromatographic column for analysis.

The analyses were performed in a CLAE system (Shimadzu, SCL 10AT VP, Japão) and coupled to the diode-array detector (DAD) (Shimadzu, SPD-M10A). It was used a RP-18 column (Phenomenex, Gemini, 250 x 4.6 mm, 5 *μ*m) and equipped with guard column (Phenomenex, ODS, 4 mm ×3 mm); mobile phase composed of methanol: ethyl acetate: acetonitrile (70 : 20 : 10 *v*/*v*/*v*), flow of 2.0 ml min^−1^, was used. Chromatograms were obtained at 450 nm [31].

The vitamin A value was calculated considering that 1 retinol activity equivalent (RAE) corresponds to 1 *μ*g retinol, 12 *μ*g of *β*-carotene, and 24 *μ*g of other provitamin A carotenoids [32].

### 2.4. Identification and Quantification of Carotenoids and Vitamin E

Compounds were identified by injecting a mixture of carotenoid and vitamin E standards and then comparing the retention times obtained for the standards and for the samples analyzed under the same conditions. In addition, vitamin E components were identified by means of chromatography and carotenoids by comparing the absorption spectra of the patterns and peaks of interest in the samples and analyzed under the same conditions, using DAD.

For the quantification of compounds, external standardization curves were used. Appropriate dilutions of the standard solutions were performed in order to obtain concentrations comparable to the levels found in the evaluated vegetables. The construction of the analytical curves was performed by injecting, in duplicate, six increasing concentrations of standard solutions. The quantification of the compounds in the samples was performed based on the analytical curves and regression equations obtained for the compounds identified in the samples: *α*-carotene (*y* = 112.991,1393*x* − 1.129,0618; *R*^2^ = 0.999), *β*-carotene (*y* = 8.039.115,2247*x* − 17.990,7687; *R*^2^ = 0.999), *α*-tocopherol (*y* = 75.930.901,9000*x* − 66.082,6598; *R*^2^ = 0.999), *α*-tocotrienol (*y* = 29.052.318,8274*x* − 106.003,6840; *R*^2^ = 0.9968), *β*-tocopherol (*y* = 78.340.650,0111*x* − 83.711,5802; *R*^2^ = 0.998), *γ*-tocopherol (*y* = 98.679.794,3633*x* − 154.659,8232; *R*^2^ = 0.997), and *δ*-tocotrienol (*y* = 142.437.744,1691*x* − 246.479,6436; *R*^2^ = 0.999).

### 2.5. Mineral Determination

To determine the minerals (Ca, Mg, Cu, Mn, Fe, Zn, Cr, Na, Se, K, and Mo), all glassware used was previously demineralized in a 10% nitric acid solution for 12 hours and dried in an air circulation oven [33]. Next, 1 g of sample and 10 ml of nitric acid were added to the digestion tubes. Afterwards, the tubes were heated in a digester block with exhaustion at an initial temperature of 80°C and a progressive raise up to 160°C, remaining at this temperature for a period of 16 hours until the formation of a clear solution. The tubes were cooled to room temperature (approximately 28°C ± 2°C), and the contents were transferred to volumetric flasks and completed up to 50 ml with deionized water. The tubes were washed with deionized water and vortexed, and their contents were poured into volumetric flasks, until the volume was completed [33]. The obtained solution was used to read the concentration of minerals through inductively coupled plasma atomic emission spectrometry (ICP-AES) (Perkin Elmer, Optima 8300).

### 2.6. Data Analysis

A completely randomized design was used with three treatments represented by each food species of the genus *Sonchus* (*S. oleraceus*, *S. asper*, and *S. arvensis*), five replicates for vitamin E and carotenoids, three replicates for proximate composition, and three replicates in duplicates for minerals. The data were stored in spreadsheets using the Microsoft Office Excel program, version 2007. To verify the existence of differences between the nutritional value of vegetables, the data were subjected to analysis of variance. To compare the means of the treatments that showed differences, the Duncan test was used, at 5% level of probability. The statistical analysis was performed using the SAS software, version 9.2 (2008), licensed to UFV.

## 3. Results and Discussion

### 3.1. Macronutrients and Caloric Density

The species analyzed in this study were collected in a wild environment, and because they are spontaneous plants, they may show high genetic variability [34]. According to Kinupp and Barros [35], little information about the nutritional composition of native food plants in Brazil is available, and when available, most are studies carried out in other countries for widely distributed species, but under very different edaphoclimatic conditions. In addition, it is highlighted that the composition of nutrients in food is influenced by inherent (age, maturity, species, variety, and form of cultivation) and environmental (climate, soil type, rainfall, and season) factors, period, and conditions of storage and handling (preparation and processing methods) [35]. Thus, comparison with other studies is rather difficult, and in the same study, considering the same species, there may be greater variability of the data between the replicates used.

Regarding the items analyzed in Table 1, the literature has few similar studies on these species, which makes it very difficult to compare them with other studies. However, it is worth mentioning that the species *S. oleraceus* is included in the Brazilian Table of Food Composition [36], with results of moisture (90.2 g/100 g), total dietary fiber (3.5 g/100 g), and ash (1.4 g/100 g), which are similar to those found in this study. This species was also analyzed by Agea et al. [37], with samples collected in Uganda and a methodology similar to this study, with results for moisture (86.32 g/100 g) and total dietary fiber (3.57 g/100 g), similar to those found here. The concentrations of macronutrients, fibers, and caloric value of the species *S. asper* and *S. oleraceus* were investigated by Guil-Guerrero et al. [38] in a study carried out in Spain. These authors found some values close to those of this study, as well as moisture (87.2 g/100 g), lipids (0.8 g/100 g), and total dietary fiber (35.6 g/100 g) in *S. oleraceus* and moisture (86.4 g/100 g) and total dietary fiber (32.5 g/100 g) in *S. asper*.

Regarding the statistical differences observed in the means of the treatment, only the moisture concentration did not differ. At the same time, caloric density was the only parameter that differed statistically in the three analyzed species. As for the other nutrients, at least in two species, there was statistical similarity, which may be related to the botanical aspect as the species belonged to the same genus, and also to the physiology of the species, verified in the physical similarity between them (Figure 1) and also shown by Khan et al. [39] and Liu et al. [40]. In the cases in which they differed, although these differences are not excessively accentuated, however, they may be associated with the fact that the samples were obtained from wild species, which have high genetic variability, and the conditions under which they developed were not subjected to control of environmental factors [34, 41].

In the few studies found on the concentration of macronutrients, fibers, and caloric value in these species, it is observed that the concentrations of moisture and dietary fibers tend to approximate, while the other items (ashes, lipids, carbohydrates, and proteins) tend to present greater variability. Although it is not the objective of this study, it is assumed that these differences may be related to several factors (technical, environmental, temporal) [37, 38, 42], since the experiments were carried out in places with different environmental characteristics and different times and in some cases with different extraction and methods of analysis.

### 3.2. Carotenoids and Vitamins

In relation to the concentration of carotenoids and vitamins in wild vegetables, besides the environmental factors interfering in their composition, the ways in which samples are obtained from the collection point to the place of processing can also interfere in the concentration of these nutrients [43]. Thus, standardization of the methodology was carefully observed as it can be seen in the methodology described.

**Figure 2:**
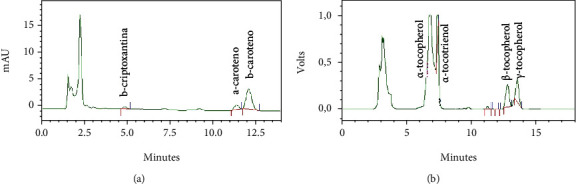
Chromatographic profile of carotenoids (a) and vitamin E (b) in *S. arvensis*.

According to the results shown in Table 2 and Figure 2, a small variability was observed between the results found for the three species. The concentrations of total carotenoids and vitamin A showed low standard deviations among the replicated. For total vitamin E, the three species differed (*α* < 0.05) in terms of concentration, in which *S. arvensis* showed the highest concentration, due to the higher concentration of *α*-tocopherol in this species.

The vitamin A values found for *S. oleraceus* in this study proved to be significant and are lower than those available in the Brazilian Table of Food Composition for this species (567 RAE/100 g) [36]. The concentration of *β-carotene* in *S. oleraceus* is also lower when compared to that found by Agea et al. [37] (13.4 mg/100 g), which is around three times higher than that observed in this study.

In our experiment, the results were inferior to those found by Guil-Guerrero et al. [38] in which *S. asper* showed 8 mg/100 g, while *S. oleraceus* showed a concentration around three times as higher when compared with the present study (15.8 mg/100 g).

Regarding the content of vitamin E, the results found in the present study were below those found in *S. asper* (49 *μ*g/100 g) by Oliveira [44]. The *α-tocopherol* content was also higher in *S. oleraceus* (197 *μ*g/100 g) and *S. asper* (205 *μ*g/100 g); although in this study, these compounds were analyzed in dry matter. The place of collection, as well as the ways in which these species are obtained, can influence the content of vitamins and minerals in vegetables, which may explain this variation between the present study and the comparative literature.

It is worth mentioning that the results found for carotenoids and vitamin E, in addition to being significant, are important from a nutritional point of view, since there are studies that prove nutritional deficiency of these compounds in the study region, so that these species can be included in food diets for children and lactating women [45, 46].

### 3.3. Minerals

The studied minerals were found in the three species investigated in this work (Table 3). However, the concentration of minerals in the foods is not a safe indicator of the nutritional value of the amounts absorbed and subsequently used by the body, since some minerals such as calcium, iron, zinc, copper, and magnesium can form insoluble complexes with antinutritional factors (phytate, oxalate) normally found in vegetables, thus decreasing their bioavailability [47].

The results found in the concentration of minerals in *S. oleraceus* are in accordance with some minerals available in the Brazilian Food Composition Table, which in turn is based on the Food Recommendations Guide of the World Health Organization (WHO), such as Mg (30 mg/100 g) and Cu (0.20 mg/100 g) [36]. Guil-Guerrero et al. [38] found *K* values for *S. asper* equal to 583.9 mg/100 g and Fe for *S. oleraceus* equal to 37.8 mg/100 g, both relatively close to those found in this study. On the other hand, in a few other studies found on the concentration of minerals in these species, there is a considerable disparity between some results. For example, the concentrations of Mg in *S. asper* and in *S. oleraceus* (0.69 mg/100 g and 0.61 mg/100 g, respectively), found by Jimoh et al. [48], are around 50 times as lower than those found in the present study. This difference may be related to the precision with which these values are expressed, in small units (mg and *μ*g). In addition, any sampling error may result in a final variable of greater amplitude. Another possibility may be related to the fertility of the soils where these plants grow, and in turn, to the place where the samples are collected, since the underground characteristics directly influence the acquisition of nutrients by the plants [49].

*S. arvensis* was the species that showed the highest concentration of *K*, while *S. asper* was the richest in Ca, and *S. oleraceus* showed the highest concentration of Fe (*α* < 0.05). Among the species analyzed in the present study, *S. arvensis* is the least studied as to its nutritional composition, although it occurs in several regions of the world besides being for food and herbal purposes [14, 50]. However, studies on its nutritional components are scarce, which values and highlights the novelty of the results found in the present study.

## 4. Conclusions

The species studied here showed an important concentration of dietary fibers and low caloric density, which is a good indicator of nutritional quality, considering that the higher the caloric density of the food ingested, the greater the risk to develop obesity, cardiovascular diseases, and so on.

The three species of the *Sonchus* genus showed carotenoids and vitamin E, where the species *S. arvensis* stood out, being the richest in vitamin E.

The minerals (K, Ca, Mg, Cu, Mn, Fe, Zn, Na, and Se) were found in the three species in the study. *S. arvensis* was the species with the highest content of potassium, while *S. asper* was the richest in calcium and *S. oleraceus* the richest in iron. Thus, the potential that these species represent for food and nutritional security strategies is emphasized, due to their wide availability in wild environments, which can be obtained with no financial costs, due to the sanity that they present as pesticides in their production systems and because they are part of the eating habits of many families and rural populations living in these regions.

## Figures and Tables

**Table 1:** Mean values of macronutrients and caloric density in *Sonchus* species.

Variables	*S. oleraceus*	*S. asper*	*S. arvensis*
Moisture (g/100 g)	91.83 ± 0.25^a^	91.77 ± 0.76^a^	92.31 ± 0.66^a^
Total dietary fiber (g/100 g)	3.97 ± 0.05^b^	4.38 ± 0.34^a^	4.30 ± 0.02^a^
Ash (g/100 g)	1.06 ± 0.02^b^	1.12 ± 0.01^a^	1.16 ± 0.02^a^
Lipids (g/100 g)	1.12 ± 0.01^b^	1.32 ± 0.08^a^	1.06 ± 0.05^b^
Carbohydrates (g/100 g)	0.17 ± 0.04^b^	0.34 ± 0.07^a^	0.12 ± 0.03^b^
Proteins (g/100 g)	1.88 ± 0.01^a^	1.97 ± 0.02^a^	1.55 ± 0.02^b^
Caloric density (kcal/100 g)	18.31 ± 0.25^b^	21.13 ± 0.91^a^	16.29 ± 0.62^c^

Values expressed in dry matter; mean of 3 replicates; data shown in mean ± standard deviation. Means followed by the same letter on the line do not differ by the test of Duncan at the 5% level of significance.

**Table 2:** Concentrations of carotenoids and vitamins in *Sonchus* species.

Compounds	*S. oleraceus*	*S. asper*	*S. arvensis*
Total vitamin A (RAE/100 g)	427.61 ± 57.03^a^	455.89 ± 39.28^a^	369.83 ± 2.84^b^
Total carotenoids (mg/100 g)	5.28 ± 0.68^b^ (100%)	5.58 ± 0.48^a^ (100%)	4.59 ± 0.03^c^ (100%)
*α-Carotene*	0.30 ± 0.02^a^ (5.69%)	0.22 ± 0.03^b^ (3.90%)	0.34 ± 0.05^a^ (7.26%)
*β-Carotene*	4.97 ± 0.78^b^ (94.31%)	5.42 ± 0.46^a^ (96.09%)	4.34 ± 0.07^a^ (92.74%)
Total vitamin E (*μ*g/100 g)	40.37 ± 3.79^c^ (100%)	49.62 ± 7.25^b^ (100%)	72.98 ± 2.91^a^ (100%)
*α-Tocopherol*	20.84 ± 0.94^b^ (51.63%)	17.02 ± 1.59^b^ (34.30%)	52.36 ± 0.40^a^ (71.75%)
*α-Tocotrienol*	13.53 ± 2.53^b^ (33.51%)	21.58 ± 4.51^a^ (43.49%)	11.26 ± 1.21^b^ (15.43%)
*β-Tocopherol*	nd	4.04 ± 0.08^a^ (8.14%)	3.75 ± 0.11^b^ (5.14%)
*γ-Tocopherol*	6.00 ± 0.32^b^ (14.86%)	6.98 ± 1.07^a^ (14.07%)	5.61 ± 1.19^c^ (7.68%)

Values expressed in fresh matter; mean of 5 replicates; data shown in mean ± standard deviation. Means followed by the same letter on the line do not differ by the test of Duncan at the 5% level of significance.

**Table 3:** Mineral concentrations in *Sonchus* species.

Minerals (mg/100 g)	*S. oleraceus*	*S. asper*	*S. arvensis*
K	490.09 ± 46.36^c^	519.34 ± 23.5^b^	604.85 ± 59.95^a^
Ca	83.26 ± 7.16^b^	96.25 ± 6.92^a^	81.91 ± 10.28^b^
Mg	30.57 ± 2.71^c^	33.34 ± 3.63^b^	38.06 ± 1.51^a^
Cu	0.13 ± 0.02^a^	0.14 ± 0.01^a^	0.12 ± 0.03^a^
Mn	0.77 ± 0.04^a^	0.83 ± 0.06^a^	0.59 ± 0.05^b^
Fe	23.74 ± 0.8^a^	7.77 ± 2.07^b^	3.43 ± 1.31^c^
Zn	0.27 ± 0.02^b^	0.43 ± 0.05^a^	0.26 ± 0.04^b^
Cr	nd	0.01 ± 0.01^a^	0.02 ± 0.00^a^
Na	5.62 ± 2.19^a^	4.81 ± 0.42^b^	4.06 ± 0.30^b^
Se	0.04 ± 0.01^a^	0.03 ± 0.01^a^	0.04 ± 0.01^a^

Values expressed in dry matter; mean of 3 replicates; data shown in mean ± standard deviation. Means followed by the same letter on the line do not differ by the test of Duncan at the 5% level of significance.

## Data Availability

Data used for this research and analysis is available from the corresponding author and will be provided upon reasonable request.
